# Proformer: a hybrid macaron transformer model predicts expression values from promoter sequences

**DOI:** 10.1186/s12859-024-05645-5

**Published:** 2024-02-20

**Authors:** Il-Youp Kwak, Byeong-Chan Kim, Juhyun Lee, Taein Kang, Daniel J. Garry, Jianyi Zhang, Wuming Gong

**Affiliations:** 1 Department of Applied Statistics, Chung‑Ang University, Seoul, Republic of Korea; 2 Cardiovascular Division, Department of Medicine, Lillehei Heart Institute, University of Minnesota, 2231 6th St SE, Minneapolis, MN 55455 USA; 3 Stem Cell Institute, University of Minnesota, Minneapolis, MN 55455 USA; 4 Paul and Sheila Wellstone Muscular Dystrophy Center, University of Minnesota, Minneapolis, MN 55455 USA; 5 Department of Biomedical Engineering, The University of Alabama at Birmingham, Birmingham, AL 35233 USA

**Keywords:** Passively Parallel Reporter Assay (MPRA), Macaron Transformer, Enhancer, Expression prediction, Sequence model

## Abstract

The breakthrough high-throughput measurement of the cis-regulatory activity of millions of randomly generated promoters provides an unprecedented opportunity to systematically decode the cis-regulatory logic that determines the expression values. We developed an end-to-end transformer encoder architecture named Proformer to predict the expression values from DNA sequences. Proformer used a Macaron-like Transformer encoder architecture, where two half-step feed forward (FFN) layers were placed at the beginning and the end of each encoder block, and a separable 1D convolution layer was inserted after the first FFN layer and in front of the multi-head attention layer. The sliding *k*-mers from one-hot encoded sequences were mapped onto a continuous embedding, combined with the learned positional embedding and strand embedding (forward strand vs. reverse complemented strand) as the sequence input. Moreover, Proformer introduced multiple expression heads with mask filling to prevent the transformer models from collapsing when training on relatively small amount of data. We empirically determined that this design had significantly better performance than the conventional design such as using the global pooling layer as the output layer for the regression task. These analyses support the notion that Proformer provides a novel method of learning and enhances our understanding of how cis-regulatory sequences determine the expression values.

## Introduction

Gene expression is a fundamental process and is essential for the coordinated function of all living organisms. Predicting the expression level of a gene based on its promoter or enhancer sequences is an important problem in molecular biology, with applications ranging from understanding the regulation of gene expression to engineering gene expression for biotechnological applications [1, 2]. Recent progress and mechanistic insights have been obtained using large-scale and high-throughput massively parallel reporter assays (MPRAs), which enable the study of gene expression and regulatory elements in a high-throughput manner and the simultaneous testing of thousands to millions of enhancers or promoters in parallel [3–25]. MPRA protocols linked random or mutated sequences to unique barcodes, with each sequence-barcode pair represented in a different reporter assay vector. After delivery of the pooled vector library, barcode abundance could be subsequently quantified using next-generation sequencing (NGS) techniques [26]. MPARs enabled large scale studies of functional annotation of putative regulatory elements [3, 27], variant effect prediction [22, 23, 28, 29] and evolutionary reconstructions [26, 30, 31]. For example, STARR-seq (self-transcribing active regulatory region sequencing) was used to investigate the enhancer activities of tens of millions of independent fragments from the Drosophila genome [3]. Microarray-based or PCA-based (polymerase cycling assembly) synthesized DNA regulatory elements with unique sequence tags were used to evaluate hundreds of thousands of variants of mammalian promoters or enhancers [4–6]. Nguyen et al. systematically compared the promoter and enhancer potentials of many candidate sequences [10]. Using Gigantic Parallel Reporter Assay (GPRA), de Boer et al. measured the expression level associated with tens of millions of random promoter sequences and used these to learn cis-regulatory logic in the yeast grown in well-characterized carbon sources [14].

Machine learning methods have been developed to identify complex relationships and patterns in large scale DNA sequences (including MPRA data) that may not be apparent through conventional statistical methods. For example, convolutional neural networks (CNN) and recurrent neural networks (RNN) were used to capture the local dependences in DNA sequences and/or genomic features and predict binding affinities [1, 32, 33], chromatin features [34, 35], DNA methylation [36, 37], RBP (RNA-binding protein) binding [38–40] and gene expression levels [41]. DeepSEA employs a convolutional neural network and has been trained on genomic sequences and large-scale chromatin-profiling data [32]. Its primary function is to learn and predict the regulatory sequence code associated with the effects of chromatin alterations. DanQ employed a deep learning model that combines a 1D CNN with a bi-directional long short-term memory network to predict the function of DNA sequences [42]. DeepATT applies 1D CNN layers and bi-LSTM layers followed by attention networks to identify functional effects using DNA sequences [43]. Vaishnav et al. proposed a CNN- and Transformer-based deep learning model for predicting gene expression levels using the millions of random promoter sequence data originally introduced by de Boer et al. in their study [25]. CRMnet is a deep learning model designed for sequence-to-expression prediction, combining 1D CNN, Transformer, and U-net architectures [44].

In this study, we developed an end-to-end transformer encoder architecture, Proformer, to predict the expression values from millions of DNA sequences. The objective of this study is to design an over-parametrized Transformer architecture for large scale regression task on DNA sequences. Proformer includes a new design named multiple expression heads (MEH) to stabilize the convergence compared with the conventional average pooling heads. Proformer ranked in the 3rd place in the final standing of the DREAM challenge: predicting gene expression using millions of random promoter sequences [45]. This DREAM challenge systematically evaluated how model architecture and training strategy choices affected model performance on prediction of functional genomic regions, and provided novel insights into designing and benchmarking genomic models across different scenarios [46]. We believe that our model provides a novel method of learning and characterizing how cis-regulatory sequences determine the expression values. Codes pertaining to important analyses in this study are available from GitHub webpage: https://github.com/gongx030/dream_PGE.

## Results

### Proformer overview

**Fig. 1:**
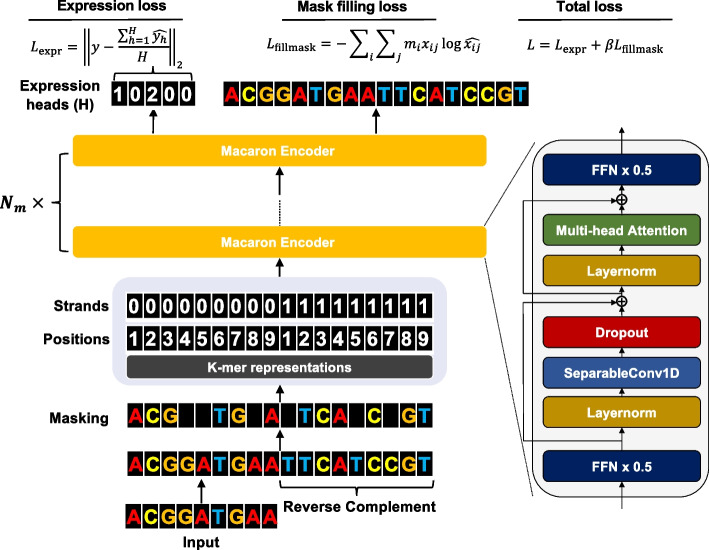
Proformer is a macaron-like transformer architecture that models the relationship between DNA sequences and expression values

The architecture of the Proformer model is described in Fig. 1. We extracted the sliding *k*-mers (*k* = 10 in the final model) from one-hot encoded sequences and mapped them onto a continuous embedding platform. It has been previously shown that the *k*-mer embedding of nucleotide sequences had better performance than the convolution on tasks such as predicting transcription factor binding sites [47]. The *k*-mer embedding was then combined with the learned positional embedding and strand embedding (forward strand vs reverse complemented strand) as one part of the input to the Macaron-like encoder.

Proformer used a Macaron-like Transformer encoder architecture to predict the expression values from promoter sequences (as shown in Fig. 1) [48–50]. Compared with the regular Transformer encoder, the Macaron-like encoder has two half-step feed forward (FFN) layers at the beginning and the end of each Transformer encoder block, which can be mathematically interpreted as a numerical Ordinary Differential Equation (ODE) solver for a convection–diffusion equation in a multi-particle dynamic system [51, 52]. Given the stochastic nature of the input sequences, we hypothesized that this design may better recover the associations between nucleotide pattern and the expression values. We added a separable 1D convolution layer in the Macaron encoder block following the first FFN layer and in front of the multi-head attention layer. This design has been used in other Transformer architectures such as Conformer [52], and is shown to be critical for capturing the local signals. The residual connections were used in each macaron block to allow the gradients to flow more directly and smoothly through the network and help prevent overfitting [48]. We iterate the Macaron-like encoder ${N}_{m}$ times.

We added $H$ positions ($H=32$ in our final model) as the expression heads (as shown in Fig. 1). Proformer predicted one expression value for each expression head and used the mean of the prediction of all positions as the final predicted expression value. The total training losses consisted of the mean squared error between predicted and observed expression values (${{\varvec{L}}}_{expr}$), and the reconstruction loss (${{\varvec{L}}}_{fillmask}$), where we randomly masked 5% of the nucleotides and had the model predict the masked nucleotides. In ${{\varvec{L}}}_{expr}$, $\widehat{{y}_{h}}$ is $h$ In Equation ${{\varvec{L}}}_{expr}$, $\widehat{{y}_{h}}$ represents the prediction made by the $h$ -th prediction head for $y$ ($h=1, \dots , H$). In ${{\varvec{L}}}_{fillmask}$, ${x}_{ij}$ denotes the value at the $i$-th position of the $j$-th sequence data. The variable ${m}_{i}$ is a mask filling indicator that ${m}_{i}=1$ indicates the i-th base of the sequence is masked. Finally, the total loss (${\varvec{L}})$ is defined as sum of ${{\varvec{L}}}_{expr}$ and ${{\varvec{L}}}_{fillmask}$ with weight $\beta$. In our final model, we set the $\beta =1$.

The final Proformer model had approximately 47 million trainable parameters, implemented by TensorFlow 2 and trained on one machine with four A100 GPUs. We varied the learning rate over the course of training according to the formula used in the original Transformer paper [48]. Warmup steps of 12,500 and a batch size of 512 were used in the training. We used the Adam optimizer with ${\beta }_{1}=0.9$, ${\beta }_{2}=0.98$ and $\epsilon ={10}^{-9}$ for these studies.

### MEH with mask filling has improved performance using large over-parameterized models

**Fig. 2:**
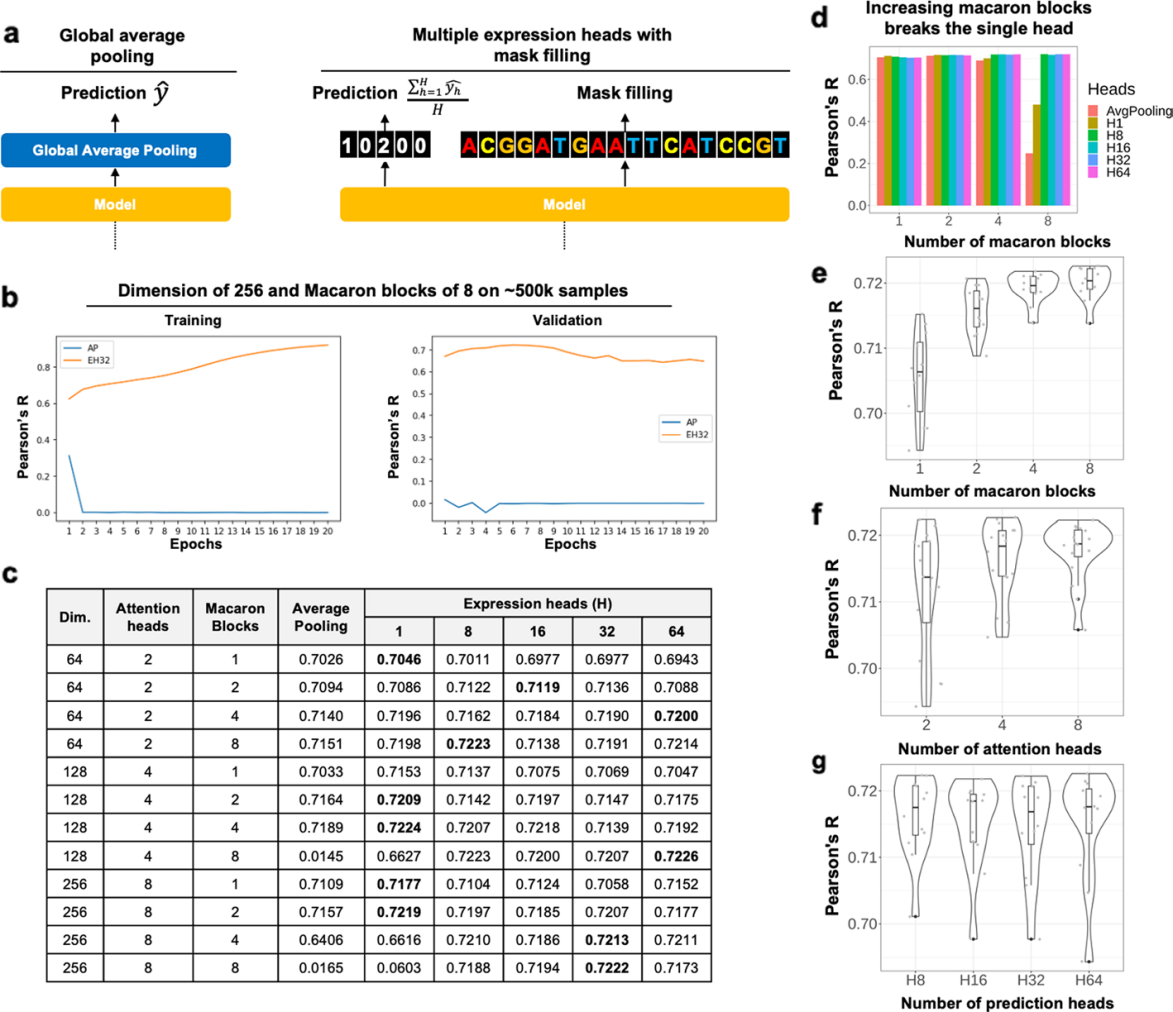
Multiple expression heads (MEH) with mask filling has better performance on large over-parameterized models. **a** Global average pooling layer and MEH with mask filling were used at the top the transformer blocks. **b** The training (left) and validation (left) performance of Proformer models using global average pooling (AP) or MEH with 32 heads (EH32) were compared. The performance was measured by the Pearson's R between observed and predicted expression values. **c** Systematic evaluation of global average pooling and MEH with mask filing on different model specifications such as dimension heads (2, 4, and 8), macaron blocks (1, 2, 4, and 8), and number of expression heads (1, 8, 16, 32, 64) was performed. The best performance of each model specification was highlighted. **d** The bar plots show the Pearson correlation between predicted and observed expression levels on the testing data. The “AvgPooling” represents a simple head, while the heads denoted by names starting with “H” incorporate multi-head and mask filling mechanisms. **e–g** The violin plots show the Pearson correlation between predicted and observed expression levels on the testing data over different number of **e** macaron blocks, **f** number of attention heads, and **g** number of prediction heads

Global average pooling layer at the top of a neural network is commonly used for the regression and classification tasks [53]. However, we found that when applying the global average pooling layer at the top of a large transformer model, for example, with a dimension size of 256 and blocks size of 8, the whole model sometimes failed to converge on training on relatively small amount (~ 500 k) of samples (Fig. 2b). To address this issue, we proposed a new design, where the model predicted multiple expression values through multiple expression heads (MEH) and used the average of all predictions as the final predicted value (Fig. 2a), while at the same time, the model also predicted the randomly masked DNA nucleotide. MEH with mask filling produced stable convergence when training the transformer model with the same size on ~ 500 k samples (Fig. 2b). To systematically compare the performance of two designs, we trained the models on 10% of the training sequence / expression value pairs then the performance was evaluated on 2% of the data as the Pearson's R between observed and predicted expression values. For MEH with mask filling, we also examined the performance over a different number of heads ($H=\mathrm{1,8},\mathrm{16,32,64}$). Overall, we found that MEH with mask filing gave significantly better than global average pooling when using 8 or more heads (Mann–Whitney U test *p* values = 0.0715, 0.0102, 0.0142, 0.0224 and 0.00605 for $H=\mathrm{1,8},\mathrm{16,32,64}$, respectively), and the best performance was achieved at a dimension size of 128 and macaron block size of 8 (Fig. 2c). As the model size became larger and deeper, the global average pooling became difficult to converge, while in comparison, MEH with masking filing could still provide stable results. Figure 2d depicts the Pearson correlation results on the test set based on the number of macaron blocks and the type of heads in the Proformer model. The “AvgPooling” represents a simple head, while the heads denoted by names starting with “H” incorporate multi-head and mask filling mechanisms. The number following “H” indicates the number of prediction heads. From the illustration, it is evident that as the count of macaron blocks increases, the performance of the model with a single head deteriorates in terms of Pearson correlation. However, employing eight or more multiple heads can rectify this issue. Figure 2e–g present a Violin plot of Pearson correlations with respect to the number of Macaron Blocks, excluding situations of ‘Average Pooling’ and ‘H1’ based on the results from Table in Fig. 2c, while displaying embedded boxplots and representing raw data as gray dots. From Fig. 2e, as the number of Macaron Blocks increases, Pearson correlation also increases. A Wilcoxon rank-sum test reveals statistically significant differences among pairs, except for cases with block counts of 4 and 8. The increase in the number of Macaron Blocks from 1 to 2, and from 2 to 4, exhibited statistically significant performance improvement (Wilcoxon rank-sum test, *p* < 0.05). Although the increase from 4 to 8 did not show statistical significance, a slight rise was observed in mean values, shifting from 0.7192 to 0.7199. From Fig. 2f, the increase in the number of attention heads (embedding dimension) from 2 (64) to 8 (256) exhibited statistically significant performance improvement (Wilcoxon rank-sum test, *p* < 0.05). While other comparisons did not exhibit statistically significant differences, on average, they showed Pearson values of 0.7117, 0.7162, and 0.7175 respectively. As evident in Fig. 2g, multiple prediction heads improved model convergence, but increasing the number of heads beyond 8 had no discernible impact on performance.

### MEH with mask filling has better performance for the prediction of chromatin accessibility from DNA sequences

**Fig. 3:**
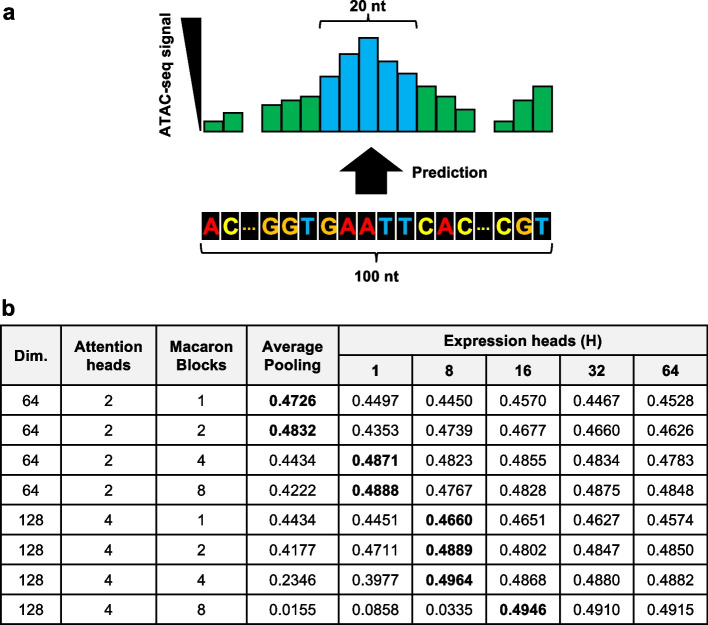
Multiple expression heads (MEH) with mask filling have better performance on predicting chromatin accessibility from DNA sequences. **a** The task of predicting mean ATAC-seq signal of the central 20 bp from 100 nt surrounding DNA sequences was examined. **b** Systematic evaluation of global average pooling and MEH with mask filing on different model specifications such as dimension heads (2 and 4), macaron blocks (1, 2, 4, and 8), and number of expression heads (1, 8, 16, 32, 64). The best performance of each model specification was highlighted

To test whether our observations on these two head designs could apply to similar scenarios, we designed another task to use Proformer to predict ATAC-seq (Assay for Transposase-Accessible Chromatin with high-throughput sequencing) signals from DNA sequences. The ATAC-seq is a technique to measure the chromatin accessibility across the whole genome [54]. We sampled a total of 100 k genomic sub-regions surrounding the ~ 80,000 summits of ATAC-seq data of GM12878 [54], while each genomic sub-region included 100 nucleotides. Different models were built to predict the mean ATAC-seq signal of the central 20 bp from 100 nt DNA sequences (Fig. 3a). The global average pooling performed well when the model size was relatively small. As the model size became larger, we observed similar trends such that the global average pooling tended to fail on large over-parameterized models. The best performance was achieved by using MEH with mask filling with dimension size of 128 and block size of 4 (Fig. 3b).

### MEH with mask filling is critical for improving the prediction performance on hold-out validation data

**Fig. 4:**
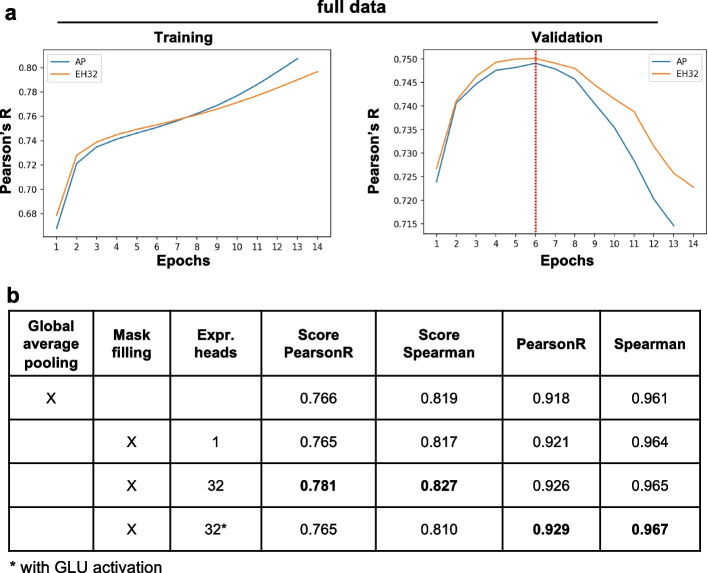
Multiple expression heads (MEH) with mask filling is critical for improving the prediction performance on hold-out validation data. **a** The training (left) and validation (left) performance of Proformer models on the full DREAM dataset using global average pooling (AP) or MEH with 32 heads (EH32). The performance was measured by the Pearson's R between observed and predicted expression values. The checkpoint after the 6th epoch was used as the final model where the validation Pearson's R was maximized (red dotted line). **b** The performance of Proformer model on the hold-out validation data. The performance is measured by weighted (score) or unweighted Pearson's R and Spearman's Rho between observed and predicted expression values. The number of Macaron blocks, number of attention heads and embedding dimension are 4, 8 and 512, respectively

We trained the final model for the DREAM challenge by using a dimension size of 512 and a block size of 4 on 95% of the data provided by the organizers and evaluated on the remaining 5%. The checkpoint after the 6th epoch was used where the validation Pearson's R was maximized. As expected, MEH with mask filling produced improved Pearson's R than global average pooling on the validation data (Fig. 4a). The ablation study showed when using only one expression head ($H=1$), the performance was similar to global average pooling. However, MEH with $H=32$ showed improvement over hold-out validation data and produced the highest weighted scores. It is interesting that adding a GLU activation [55] to expression heads produced even higher unweighted Pearson’s R and Spearman's Rho on the hold-out validation data, while the weighted score became worse than global average pooling (Fig. 4b). Future studies will explore different designs of the expression heads.

### Proformer outperforms other methods on the random promoter dataset

**Fig. 5:**
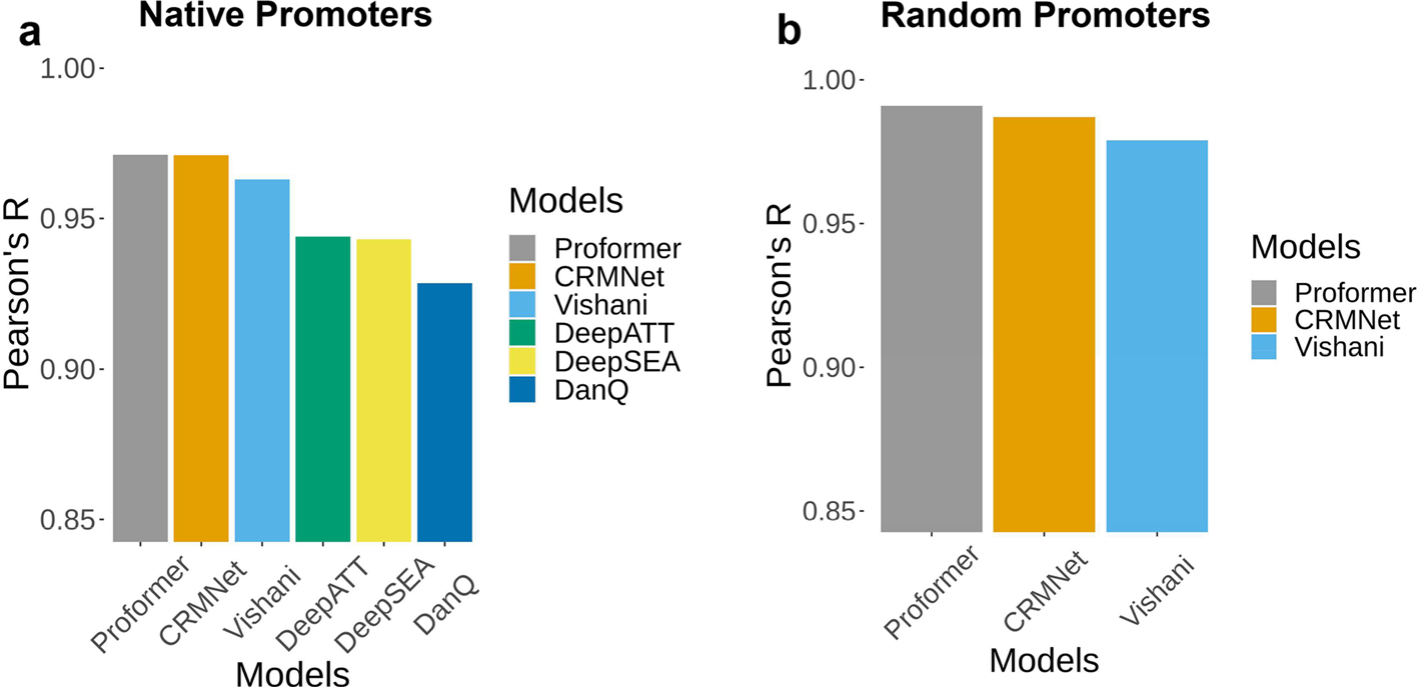
Performance comparison between the Proformer model and existing models: The prediction performance was evaluated on yeast native promoters and random promoters, and compared with DeepSEA, DanQ, DeepAtt, CRMnet, and the transformer model proposed by Vaishnav et al. (2022). The performance metrics for CRMnet, DeepAtt, DanQ, and DeepSEA were obtained from Vaishnav et al. (2022) and Ding et al. (2023). **a** In the case of the Native Promoter dataset, our proposed Proformer model exhibited performance nearly equivalent to that of the CRMNet model. **b** For the Random Promoter dataset, our Proformer model outperformed all other models, demonstrating superior performance

To facilitate a comparative analysis with other existing models, we conducted experiments with the Proformer model on the dataset used by Vaishnav et al. [25]. In total, there were 51,339,035 samples in the training data, and for evaluation, we had expression level data for 61,150 yeast native promoter sequences and 2,954 random promoter sequences. We trained the Proformer model on the training data and calculated the Pearson's R value on the evaluation data for comparison with other methods. The performance of other existing methods was obtained from Vaishnav et al. and Ding et al. [25, 44]. Figure 5 presents a bar plot comparing the Pearson's R correlation values of various existing models. As observed in Fig. 5a, for the native promoter dataset, both the Proformer model and the CRMNet model exhibited similar performance, with R values of 0.971. Vaishnav et al.'s Transformer model achieved the third-best performance with an R value of 0.963. On the random promoter dataset, the Proformer model outperformed all others with an R value of 0.991. CRMNet and Vaishnav et al.'s Transformer model achieved R values of 0.987 and 0.979, respectively (Fig. 5b).

## Discussion

Various machine learning techniques have been used to analyze and interpret the MPRA data and dissect the regulatory logics. Recently, over-parameterized deep networks or large models, with more parameters than the size of the training data, have dominated the performance in various machine learning areas [56]. The global average pooling layer was conventionally used to aggregate the information from multiple channels and to produce final predictions. However, we found that when training over-parameterized models on the regression tasks such as predicting expression values from DNA sequences, the global average pooling often led to a convergence issue, most likely due to the loss of information that accumulated during the training and caused the model to perform poorly or failed to converge. Here we presented a new architecture Proformer for prediction of expression values from DNA sequences. We introduced a new design named multiple expression heads (MEH) with mask filling to prevent the over-parameterized transformer models from collapsing when training on relatively small amount of data. Applying the Proformer model to predict expression values and to predict chromatin accessibility from DNA sequences showed that MEH with masking filling produced significantly better performance and stable convergence compared to the commonly used global average pooling. This suggests that MEH with mask filling could be an effective design strategy for similar regression tasks in genomics, especially when utilizing large, over-parameterized models. For future research directions, it would be beneficial to explore the development of a versatile pre-trained network for large-scale DNA data. Similar to how language models are fine-tuned for specific tasks in natural language processing, pre-training large DNA models on extensive DNA datasets and subsequently applying them to specific tasks could be an intriguing avenue. MEH with mask filling provides a new strategy of pre-training large DNA sequence models. Additionally, investigating the application of pre-trained models in genomics, utilizing transfer learning from existing DNA data, holds promise as an exciting research topic.

## Methods

### DREAM challenge dataset overview

Rafi et al. conducted a high-throughput experiment to measure the regulatory effect of millions of random DNA sequences. They cloned 80 bp random DNA sequences into a promoter-like context upstream of a yellow fluorescent protein (YFP), transformed the resulting library into yeast, and measured expression by fluorescent activated cell sorting [4, 14, 57]. The training dataset includes 6,739,258 random promoter sequences and their corresponding mean expression values [45].

Rafi et al. also provided 71,103 sequences from several promoter sequence types as the hold-out "validation" dataset to evaluate the model performance in different ways. These validation datasets included predicting the expression changes resulting from single nucleotide variants (SNVs), perturbation of specific transcription factor (TF) binding sites, tiling of TF binding sites across background sequences, sequences with high- and low-expression levels, native yeast genomic sequences, random DNA sequences, and challenging sequences designed to maximize differences between a convolutional model and a biochemical model trained on the same data [45].

Training the model using the entire dataset on a single A100 GPU requires a significant amount of time, approximately one day. To reduce the time required, during the hyperparameter tuning process, we trained the model on a randomly selected 10% subset of the training data and used approximately 2% of the data as a validation set.

### Sequence trimming and padding

We removed the leading 17 and trailing 13 nucleotides (nt) that were identical in both training and testing promoter sequences, since these nucleotides were not informative for the prediction of expression values and removal of the nucleotides would significantly reduce the training and inference time. The length of the resulting promoter sequences ranged from 48 to 112 nt for training data, while > 99.97% training promoters were less than 100 nucleotides. To further reduce the computational overhead, we used 6,737,568 promoter sequences shorter than 100 nt (after trimming) in the model training. For promoters that were less than 100 nt, the left and right sides were padded with the letter N.

### Reverse complemented sequences

We empirically found that including the reverse complemented promoter sequences would significantly improve the performance. Thus, the reverse complemented sequences were concatenated with the original sequences (after trimming and padding) and used as the input for model training. Thus, the total length of the input sequences was 200 nt.

### Standardization of the expression values

The expression values were standardized to the mean of zero and standard deviation of one. Our experiments found that when the mean squared error loss was used, standardizing the expression values gave better model generalization performance (in terms of Pearson's R and Spearman's Rho) and faster convergence.

### Evaluation metric

Our primary evaluation metric was the Pearson's R correlation between the actual expression levels and the predicted expression levels generated by the model. Given the slight differences in the settings between the training and evaluation datasets, utilizing this metric was meaningful in preserving the order of results. It's worth noting that similar studies conducted by Vaishnav et al. and Ding et al., using comparable datasets, also employed Pearson's R correlation as their primary evaluation metric.

### Vaishnav dataset overview

The Vaishnav dataset closely resembles the data from the DREAM challenge. We employed data obtained from cells cultured in both the complex medium (containing yeast extract, peptone, and dextrose) and the defined medium (lacking uracil) as specified in the study by Vaishnav et al. [25]. A total of 30,722,376 sequences were obtained from the complex medium, while 20,616,659 sequences originated from the defined medium. This dataset was utilized for comparative analysis with several other methods. For the evaluation dataset, there were 61,150 native promoter sequences and 2,954 random promoter sequences.

### ATAC-seq data proeprocessing

The human EBV-transformed lymphoblastoid cell line (LCL) ATAC-seq data were downloaded from NCBI GEO database (GSE47753). The sequence reads from three replicates of 50 k cell sample (GSM1155957, GSM1155958 and GSM1155959) were pooled and used for the downstream analysis. The sequencing reads where mapped to the mouse genome (mm10) using Bowtie 2 [58]. The ATAC-seq lied within the blacklisted genomic regions for functional genomics analysis were excluded [59]. The 86,004 peaks called by MACS2(v2.1.1) [60, 61] were used for the downstream analysis. We randomly sampled a total of 100 k genomic sub-regions surrounding each summit of ATAC-seq data of GM12878 [54], while each genomic sub-region included 100 nucleotides. We built models to predict the mean ATAC-seq signal of the central 20 bp from each 100 nt DNA sequences. We randomly used 90% (90 k) sequences for training and 10% (10 k) sequences for testing.

## Data Availability

The source code and datasets used to obtain the results presented in this article are available on GitHub (https://github.com/gongx030/dream_PGE) under the GNU 3 license. The raw dataset for the DREAM challenge can be found at https://www.synapse.org/#!Synapse:syn28469146/wiki/617075. The ATAC-seq data for GM12878 can be at NCBI GEO database with accession GSE47753.

## References

[1] Alipanahi B, Delong A, Weirauch MT, Frey BJ (2015). Predicting the sequence specificities of DNA- and RNA-binding proteins by deep learning. Nat Biotechnol.

[2] Bussemaker HJ, Foat BC, Ward LD (2007). Predictive modeling of genome-wide mRNA expression: from modules to molecules. Annu Rev Bioph Biom.

[3] Arnold CD, Gerlach D, Stelzer C, Boryń ŁM, Rath M, Stark A (2013). Genome-wide quantitative enhancer activity maps identified by STARR-seq. Science.

[4] Sharon E, Kalma Y, Sharp A, Raveh-Sadka T, Levo M, Zeevi D (2012). Inferring gene regulatory logic from high-throughput measurements of thousands of systematically designed promoters. Nat Biotechnol.

[5] Melnikov A, Murugan A, Zhang X, Tesileanu T, Wang L, Rogov P (2012). Systematic dissection and optimization of inducible enhancers in human cells using a massively parallel reporter assay. Nat Biotechnol.

[6] Patwardhan RP, Hiatt JB, Witten DM, Kim MJ, Smith RP, May D (2012). Massively parallel functional dissection of mammalian enhancers in vivo. Nat Biotechnol.

[7] Kheradpour P, Ernst J, Melnikov A, Rogov P, Wang L, Zhang X (2013). Systematic dissection of regulatory motifs in 2000 predicted human enhancers using a massively parallel reporter assay. Genome Res..

[8] Lubliner S, Regev I, Lotan-Pompan M, Edelheit S, Weinberger A, Segal E (2015). Core promoter sequence in yeast is a major determinant of expression level. Genome Res.

[9] Farley EK, Olson KM, Zhang W, Brandt AJ, Rokhsar DS, Levine MS (2015). Suboptimization of developmental enhancers. Science.

[10] Nguyen TA, Jones RD, Snavely AR, Pfenning AR, Kirchner R, Hemberg M (2016). High-throughput functional comparison of promoter and enhancer activities. Genome Res.

[11] Smith RP, Taher L, Patwardhan RP, Kim MJ, Inoue F, Shendure J (2013). Massively parallel decoding of mammalian regulatory sequences supports a flexible organizational model. Nat Genet.

[12] Mogno I, Kwasnieski JC, Cohen BA (2013). Massively parallel synthetic promoter assays reveal the in vivo effects of binding site variants. Genome Res.

[13] van Arensbergen J, FitzPatrick VD, de Haas M, Pagie L, Sluimer J, Bussemaker HJ (2017). Genome-wide mapping of autonomous promoter activity in human cells. Nat Biotechnol.

[14] de Boer CG, Vaishnav ED, Sadeh R, Abeyta EL, Friedman N, Regev A (2020). Deciphering eukaryotic gene-regulatory logic with 100 million random promoters. Nat Biotechnol.

[15] Gertz J, Siggia ED, Cohen BA (2009). Analysis of combinatorial cis-regulation in synthetic and genomic promoters. Nature.

[16] Weingarten-Gabbay S, Nir R, Lubliner S, Sharon E, Kalma Y, Weinberger A (2019). Systematic interrogation of human promoters. Genome Res.

[17] Grossman SR, Zhang X, Wang L, Engreitz J, Melnikov A, Rogov P (2017). Systematic dissection of genomic features determining transcription factor binding and enhancer function. Proc Nat Acad Sci.

[18] Shen SQ, Myers CA, Hughes AEO, Byrne LC, Flannery JG, Corbo JC (2016). Massively parallel cis-regulatory analysis in the mammalian central nervous system. Genome Res.

[19] Haberle V, Arnold CD, Pagani M, Rath M, Schernhuber K, Stark A (2019). Transcriptional cofactors display specificity for distinct types of core promoters. Nature.

[20] Klein JC, Agarwal V, Inoue F, Keith A, Martin B, Kircher M (2020). A systematic evaluation of the design and context dependencies of massively parallel reporter assays. Nat Methods.

[21] Vockley CM, Guo C, Majoros WH, Nodzenski M, Scholtens DM, Hayes MG (2015). Massively parallel quantification of the regulatory effects of noncoding genetic variation in a human cohort. Genome Res.

[22] Tewhey R, Kotliar D, Park DS, Liu B, Winnicki S, Reilly SK (2016). Direct identification of hundreds of expression-modulating variants using a multiplexed reporter assay. Cell.

[23] Ulirsch JC, Nandakumar SK, Wang L, Giani FC, Zhang X, Rogov P (2016). Systematic functional dissection of common genetic variation affecting red blood cell traits. Cell.

[24] Liu S, Liu Y, Zhang Q, Wu J, Liang J, Yu S (2017). Systematic identification of regulatory variants associated with cancer risk. Genome Biol.

[25] Vaishnav ED, de Boer CG, Molinet J, Yassour M, Fan L, Adiconis X (2022). The evolution, evolvability and engineering of gene regulatory DNA. Nature.

[26] Romero IG, Lea AJ (2023). Leveraging massively parallel reporter assays for evolutionary questions. Genome Biol.

[27] Kwasnieski JC, Fiore C, Chaudhari HG, Cohen BA (2014). High-throughput functional testing of ENCODE segmentation predictions. Genome Res.

[28] Castaldi PJ, Guo F, Qiao D, Du F, Naing ZZC, Li Y (2018). Identification of functional variants in the FAM13A chronic obstructive pulmonary disease genome-wide association study locus by massively parallel reporter assays. Am J Resp Crit Care.

[29] Shen SQ, Kim-Han JS, Cheng L, Xu D, Gokcumen O, Hughes AEO, et al. A candidate causal variant underlying both enhanced cognitive performance and increased risk of bipolar disorder. Biorxiv. 2021; 580258

[30] Klein JC, Keith A, Agarwal V, Durham T, Shendure J (2018). Functional characterization of enhancer evolution in the primate lineage. Genome Biol.

[31] Arnold CD, Gerlach D, Spies D, Matts JA, Sytnikova YA, Pagani M (2014). Quantitative genome-wide enhancer activity maps for five Drosophila species show functional enhancer conservation and turnover during cis-regulatory evolution. Nat Genet.

[32] Zhou J, Troyanskaya OG (2015). Predicting effects of noncoding variants with deep learning–based sequence model. Nat Methods.

[33] Kelley DR, Snoek J, Rinn JL (2016). Basset: learning the regulatory code of the accessible genome with deep convolutional neural networks. Genome Res..

[34] Kelley DR, Reshef YA, Bileschi M, Belanger D, McLean CY, Snoek J (2018). Sequential regulatory activity prediction across chromosomes with convolutional neural networks. Genome Res.

[35] Dsouza N, Gong W, Garry DJ. SeATAC: a tool for exploring the chromatin landscape and the role of pioneer factors. Biorxiv. 2022:2022.04.25.489439 10.1186/s13059-023-02954-5 PMC10204251 37218013

[36] Zeng H, Gifford DK (2017). Predicting the impact of non-coding variants on DNA methylation. Nucleic Acids Res..

[37] Angermueller C, Lee HJ, Reik W, Stegle O (2017). DeepCpG: accurate prediction of single-cell DNA methylation states using deep learning. Genome Biol.

[38] Pan X, Rijnbeek P, Yan J, Shen H-B (2018). Prediction of RNA-protein sequence and structure binding preferences using deep convolutional and recurrent neural networks. BMC Genom.

[39] Budach S, Marsico A (2018). Pysster: classification of biological sequences by learning sequence and structure motifs with convolutional neural networks. Bioinformatics.

[40] Avsec Ž, Barekatain M, Cheng J, Gagneur J (2018). Modeling positional effects of regulatory sequences with spline transformations increases prediction accuracy of deep neural networks. Bioinformatics.

[41] Zhou J, Theesfeld CL, Yao K, Chen KM, Wong AK, Troyanskaya OG (2018). Deep learning sequence-based ab initio prediction of variant effects on expression and disease risk. Nat Genet.

[42] Quang D, Xie X (2016). DanQ: a hybrid convolutional and recurrent deep neural network for quantifying the function of DNA sequences. Nucl Acids Res.

[43] Li J, Pu Y, Tang J, Zou Q, Guo F (2020). DeepATT: a hybrid category attention neural network for identifying functional effects of DNA sequences. Brief Bioinform..

[44] Ding K, Dixit G, Parker BJ, Wen J (2023). CRMnet: A deep learning model for predicting gene expression from large regulatory sequence datasets. Front Big Data.

[45] Rafi AM. Evaluation and optimization of sequence-based gene regulatory deep learning models. 2023.

[46] Rafi AM, Penzar D, Nogina D, Lee D, Vaishnav ED, Lee D, et al. Evaluation and optimization of sequence-based gene regulatory deep learning models. 2023. 10.1101/2023.04.26.538471

[47] Shen Z, Bao W, Huang D-S (2018). Recurrent neural network for predicting transcription factor binding sites. Sci Rep.

[48] Vaswani A, Shazeer N, Parmar N, Uszkoreit J, Jones L, Gomez AN, et al. Attention is all you need. arXiv.org. 2017;cs.CL.

[49] Press O, Smith NA, Levy O. Improving transformer models by reordering their sublayers. Arxiv. 2019.

[50] Lu Y, Li Z, He D, Sun Z, Dong B, Qin T, et al. Understanding and improving transformer from a multi-particle dynamic system point of view. Arxiv. 2019.

[51] Lu Y, Li Z, He D, Sun Z, Dong B, Qin T, et al. Understanding and improving transformer from a multi-particle dynamic system point of view. Arxiv. 2019. 10.48550/arxiv.1906.02762.

[52] Gulati A, Qin J, Chiu C-C, Parmar N, Zhang Y, Yu J, et al. Conformer: convolution-augmented transformer for speech recognition. Arxiv. 2020.

[53] Pak M, Kim S. A Review of deep learning in image recognition. In: 2017 4th international conference on computer applications and information processing technology Caipt. 2017; 1–3.

[54] Buenrostro JD, Giresi PG, Zaba LC, Chang HY, Greenleaf WJ (2013). Transposition of native chromatin for fast and sensitive epigenomic profiling of open chromatin, DNA-binding proteins and nucleosome position. Nat Methods.

[55] Dauphin YN, Fan A, Auli M, Grangier D. Language modeling with gated convolutional networks. Arxiv. 2016

[56] Liu S, Zhu Z, Qu Q, You C. Robust training under label noise by over-parameterization. Arxiv. 2022. 10.48550/arxiv.2202.14026

[57] Kinney JB, Murugan A, Callan CG, Cox EC (2010). Using deep sequencing to characterize the biophysical mechanism of a transcriptional regulatory sequence. Proc National Acad Sci.

[58] Langmead B, Salzberg SL (2012). Fast gapped-read alignment with Bowtie 2. Nat Methods.

[59] Amemiya HM, Kundaje A, Boyle AP (2019). The ENCODE blacklist: identification of problematic regions of the genome. Sci Rep-uk.

[60] Zhang Y, Liu T, Meyer CA, Eeckhoute J, Johnson DS, Bernstein BE (2008). Model-based analysis of ChIP-Seq (MACS). Genome Biol.

[61] Feng J, Liu T, Qin B, Zhang Y, Liu XS (2012). Identifying ChIP-seq enrichment using MACS. Nat Protoc.

[62] Gong W. Proformer: a hybrid Macaron transformer model predicts expression values from promoter sequences. In: 14th annual RECOMB/ISCB conference on regulatory and systems genomics with DREAM challenges, RSGDREAM 2022 10.1186/s12859-024-05645-5 PMC10877777 38378442

